# Neurological manifestations in autoinflammatory syndromes: a series of 131 patients from our neuroimmunology departement

**DOI:** 10.1186/1546-0096-13-S1-P150

**Published:** 2015-09-28

**Authors:** E Schuh, P Lohse, J Havla, I Meinl, L-A Gerdes, R Hohlfeld, T Kümpfel

**Affiliations:** 1 Institute of Clinical Neuroimmunology, Munich, Germany; 2 Labor Blessing, Singen, Germany

## Introduction

Cryopyrin-associated periodic syndrome (CAPS), familial mediterranean fever (FMF) and tumor necrosis factor receptor-associated periodic syndromes (TRAPS) are rare systemic, monogenetic inherited autoinflammatory diseases. The clinical significance of low penetrance mutations in TRAPS and CAPS as well as heterozygosity in FMF is still under debate. The frequency and clinical pattern of peripheral and central nervous system (CNS) manifestation in those patients remain unclear.

## Objective/methods

To describe the neurological phenotype of patients with a low penetrance variant in the *TNFRSF1A* gene, the *NLRP3* gene, or with a heterozygous mutation in the *MEFV* gene seen in our neuroimmunology department between 2006 and 2015.

## Results

131 patients (f=88, m=43; mean age 41 ± 10 years) have been identified in our outpatient clinic: 65 with a low penetrance mutation in the *TNFRSF1A* gene, 23 with a low penetrance variants in the *NLRP3* gene and 43 with a heterozygous mutation in the *MEFV* gene. 90 patients had an additional diagnosis of multiple sclerosis (MS). Headache and chronic pain syndromes were observed among TRAPS, CAPS and FMF patients. Inflammation predominately affected the eye and optic nerve. Otherwise the neurological phenotype was heterogeneous among groups and included mostly dysesthesia in TRAPS patients, cranial nerve (CN) affection in CAPS patients and CNS vasculitis in the FMF non-MS cohort. Other systemic symptoms included abdominal pain, arthralgias and myalgias, urticarial rash and severe fatigue. So far none of the patients had developed AA amyloidosis.

## Conclusion

In this large mono-centric cohort of patients with autoinflammatory syndromes concomitant MS was frequently observed indicating that mutations in the *TNFRSF1A, NLRP3* and *MEFV* gene may contribute to MS susceptibility. Besides MS a variety of neurological manifestations can occur and TRAPS, CAPS and FMF should be included as differential diagnosis in patients with unusual CNS inflammation including CN affection, severe headache syndromes and additional systemic symptoms such as abdominal pain, arthralgia and urticarial rash suggestive for an autoinflammatory disease.

